# Connect 4: A Novel Paradigm to Elicit Positive and Negative Insight and Search Problem Solving

**DOI:** 10.3389/fpsyg.2018.01755

**Published:** 2018-10-25

**Authors:** Gillian Hill, Shelly M. Kemp

**Affiliations:** ^1^Department of Psychology, University of Buckingham, Buckingham, United Kingdom; ^2^Learning and Teaching Institute, University of Chester, Chester, United Kingdom

**Keywords:** creative problem solving, negative insight, Aha, Uh-oh, Connect 4

## Abstract

Researchers have typically defined insight as a sudden new idea or understanding accompanied by an emotional feeling of Aha. Recently, examples of negative insight in everyday creative problem solving have been identified. These are seen as sudden and sickening moments of realization experienced as an Uh-oh rather than Aha. However, such experiences have yet to be explored from an experimental perspective. One barrier to doing so is that methods to elicit insight in the laboratory are constrained to positive insight. This study therefore aimed to develop a novel methodology that elicits both positive and negative insight solving, and additionally provides the contrasting experiences of analytic search solving in the same controlled conditions. The game of Connect 4 was identified as having the potential to produce these experiences, with each move representing a solving episode (where best to place the counter). Eighty participants played six games of Connect 4 against a computer and reported each move as being a product of positive search, positive insight, negative search or negative insight. Phenomenological ratings were then collected to provide validation of the experiences elicited. The results demonstrated that playing Connect 4 saw reporting of insight and search experiences that were both positive and negative, with the majority of participants using all four solving types. Phenomenological ratings suggest that these reported experiences were comparable to those elicited by existing laboratory methods focused on positive insight. This establishes the potential for Connect 4 to be used in future problem solving research as a reliable elicitation tool of insight and search experiences for both positive and negative solving. Furthermore, Connect 4 may be seen to offer more true to life solving experiences than other paradigms where a series of problems are solved working toward an overall superordinate goal rather than the presentation of stand-alone and un-related problems. Future work will need to look to develop versions of Connect 4 with greater control in order to fully utilize this methodology for creative problem solving research in experimental psychology and neuroscience contexts.

## Introduction

An insight moment is defined as a sudden new understanding, idea or solution accompanied by an emotional Aha experience (Jung-Beeman et al., 2008; Klein and Jarosz, 2011). Insight has long been recognized as a desirable feature of creative problem solving, with many famous examples of discoveries in STEM (Science, Technology, Engineering, and Mathematics) being attributed to it. Maryam Mirzakhani, winner of the Field’s medal demonstrates this when asked about mathematics, “the most rewarding part is the ‘Aha’ moment, the excitement of discovery and enjoyment of understanding something new, the feeling of being on top of a hill, and having a clear view" (CMI, 2008, p. 12). A similar rewarding aspect to insight moments has recently been demonstrated by Friedlander and Fine (2016) whose Cryptic Crossword solving sample identified the Penny Dropping Moment (the Crossword solver community’s term for insight moments) as the main motivation for pursuing their hobby. In both these examples the insight experience is a positive one, something that can be seen as a tacit assumption in the historical approach to insight research (Gick and Lockhart, 1995). More recently, however, it has been proposed that insight moments might incorporate negative realizations, with an accompanying Uh-oh moment rather than the prototypical Aha (Hill and Kemp, 2016; Hill and Kemp, unpublished a). This presents a problem for current methods that elicit insight for empirical exploration, which are only designed to produce positive solving experiences. Therefore the development of new methods that stimulate a full range of solving experiences is required to reflect and experimentally test these recent developments in the insight and creativity literature. As such this article describes a preliminary exploration of a new method to elicit experiences that incorporate both positive and negative insight and search solving.

Contemporary research has begun to take a renewed interest in the phenomenology of insight with a varying focus on emotional experiences (Danek et al., 2014a; Jarman, 2014). Danek et al.’s (2014a) participants attempted to solve the puzzle of how a magician had performed different tricks and demonstrated that the resulting solutions arose through both insight and search strategies. In a novel step, after they had completed all the trials participants reported their experiences whilst solving the tricks through insight using a visual analog scale (VAS) to rate against various components. The components of these scales were identified by the researchers and verified through qualitative, open solving descriptions from the participants given before they offered the ratings. Ratings were made for the level of impasse participants experienced before their Aha moment; how pleasant, sudden and surprising solutions were and; how certain they were of the insight solutions they found. Pleasantness was the highest rated feature, with impasse being interpreted as least indicative of Aha solutions. However, as recognized by Danek et al. (2014a), no ratings were recorded for search solutions meaning it was not clear if the phenomenological features identified were unique to insight solving and separable from more general responses to solving problems.

Webb et al. (2016) used the phenomenological rating scales developed by Danek et al. (2014a) across a variety of established tasks that elicit insight problem solving experience. Rather than use a dichotomous approach to labeling of solving experience (i.e., search or insight), their participants rated their feeling of Aha on a VAS (rating the feeling of Aha). They found that pleasantness was positively correlated with feelings of Aha and this effect was consistent across the different types of problem presented [classic insight, classic non-insight and Compound Remote Associates (CRA)]. Other features showed less consistency, notably impasse either showed no correlation or a negative correlation. These ratings were made on a trial-by-trial basis offering further support for the scales’ validity in capturing phenomenological components of insight. As such these studies provide converging evidence to support the importance of further exploration of the emotional component in insight using phenomenological ratings to do so.

Affective aspects of insight have been discussed historically, despite not being explored experimentally until recently. Gick and Lockhart (1995) raised the possibility that insight experiences may not be universally experienced as pleasant. They identified that some solutions might also be accompanied by chagrin, annoyance at the obviousness of the revelation they had previously missed. Hill and Kemp (2016) further explored the notion of negative aspects of insight in a qualitative study. They recorded reports of everyday, sudden realizations that did not represent the positive Aha experiences attached to solving a problem. Instead they demonstrated that negative insights, experienced as Uh-oh moments served to identify problems rather than resolve them. A notable example of this is described by software entrepreneur and philanthropist Dame Stephanie ‘Steve’ Shirley when outlining the coding process. She describes how she often identified mistakes in her computer coding as sudden negative insights that occurred early in the morning as she awoke (Al-Khalili, 2015); negative insight served to alert her to problems previously unforeseen that she would then work to solve. This demonstrates a proposed adaptive function of negative insight (Hill and Kemp, unpublished a), where identifying a problem has long been seen as an important element of problem solving (Guilford, 1951; Csikszentmihalyi and Sawyer, 1995; Runco and Chand, 1995).

However, whilst Hill and Kemp’s (2016) research demonstrates experience of negative insight in everyday context this was based on qualitative reports, which leave a number of unanswered questions. There has been little exploration of how components of the insight moment that are considered emotional and cognitive are related. Topolinski and Reber (2010) asserted that emotional components are epiphenomena, occurring after the purely cognitive insight event. In such an account the negative flavor of some insights would result from subsequent appraisals, perhaps of disappointment or frustration. However, no experimental evidence has to date been provided to directly support this. Furthermore, examination of emotion literature highlights different theoretical perspectives that challenge the assumption that cognitive events necessarily precede an emotional evaluation. For example, Barrett’s (2014) Conceptual Act Theory contends that the separation of mental processes to cognitive and emotional is a false dichotomy arguing that both are outcomes of integrated constructed experience rather than one being a consequence of the other. It positions valenced core affect as central to mental events that are then constructed as cognitive, emotional, or perceptual. By this account an insight moment would occur with intrinsic positive or negative core affect contingent on the insight context [whether the realization was ‘good for me’ or ‘bad for me’ (Gross, 2015)]. This study takes a first step to such experimental exploration through the development of a task that can provide insight moments that are both positive and negative.

The types of task typically used to elicit insight were developed against the definition of insight, which carries the tacit assumption that insight is positive and represents a solving experience (for example see Gilhooly and Murphy, 2005; Cunningham et al., 2009; Salvi et al., 2016; Webb et al., 2016). However, the phenomenological scales developed by Danek et al. (2014a) do include the potential to measure negative insight, as they range from very pleasant (scored 100) to very unpleasant (scored as 0). Yet in their original study, participants’ responses on average ranged in the positive half of the scale (well above 50), demonstrating that while the possibility to measure negative experiences is available, current paradigms do not elicit this full range of emotional insight responses. Webb et al.’s (2016) positive correlation suggests that as problems were solved with greater feeling of insight so were they generally rated more positively. However, any exceptions to this association could well be hidden by the overall trend. As such current tasks can be seen to offer limited opportunities to investigate negative insight moments that potentially occur at earlier stages of the problem solving process, for example representing sudden episodes of problem finding rather than solution finding. Therefore the full range of insight from negative to positive has yet to be fully explored through current experimental paradigms.

Current methods offer the opportunity for isolated and convergent solving experiences, with the solving moment signifying the culmination of the trial. For complex real-life problems, solving rarely happens in a single insight or search episode. Fleck and Weisberg (2013) and Weisberg (2014) proposed a model of problem solving to explain a continuum from insight to analysis when finding a solution. Within the stages of this model examples of mini-solving episodes can be seen that move the solver closer to their overall superordinate goal and may offer a model that better maps to real-life solving. In fact the subordinate, mini-solving episodes in this model might be considered as a series of problem solving events leading to an ultimate overall goal. In this context, the potential for negative insight moments can be identified, when a solving attempt fails but new information arises suddenly as a result of the failure. These Uh-oh moments initiate new problem solving efforts, perhaps in a different direction that may move the individual closer to their overall goal.

This illustrates that different levels of focus can be applied when considering problem solving, a point made by Perkins (2001) who identified a structure to break-through ideas common across different scales of problem solving. He outlined examples widening in scale from an individual’s idea in the moment (more everyday insight) to ‘great’ profound realizations resulting from a life’s work; for example Darwin’s development of the theory of evolution. In the extreme Perkins (2001) even proposed consideration of problem solving on an evolutionary timescale. Such an approach again highlights a disparity between the types of tasks currently used to explore insight problem solving in the lab. and more naturalistic, real-life solving experience. Many current methods present discrete solving episodes that are unconnected to each other, whilst solving in everyday life often sees related solving episodes moving toward an overall goal.

Table top games can be seen to mimic this, with a series of moves or turns working toward the overall goal of winning the game. Chess has been used by cognitive psychologists to explore problem solving and decision making and incorporates positive and negative experiences as a player builds a winning position and identifies potential negative threats from their opponent (Chase and Simon, 1973; Charness, 1992; Gobet and Simon, 1996; Leone et al., 2017). However, the need to learn the rules of chess and differing levels of player ability could introduce potential confounds when being used to explore problem solving behavior. A similarly dyadic game to chess, but with even simpler rules is Connect 4. Players take turns to drop counters (each player has separate colored counters) into a vertical grid, the standard version being seven positions wide and six counters deep. The counter falls to the lowest position, so the first to be dropped into a column will occupy the lowest row with subsequent counters sitting on top of each other. The winner of the game is the first to get four adjacent counters in a line; this can be horizontally, vertically, or diagonally. In playing the game both search and non-search intuitive strategies (potentially insight) can be employed to select moves (Mańdziuk, 2012). These moves like chess may be positively focused toward building a winning position or responding to a negative realization aimed at preventing an immediate loss. As such, Connect 4 would seem to be a candidate platform to elicit repeated episodes of positive and negative solving (selecting the best move) in the controlled environment of game play. These solutions being arrived at through analytic means or in an experience of insight congruent to those reported in other insight research (for example Bowden and Jung-Beeman, 2003a; Danek et al., 2014a)

Furthermore, Connect 4 with a maximum of 21 moves leading to a full grid and stalemate means that a game takes a much shorter time to play than for chess. Yet it retains the desirable features highlighted by researchers in problem solving and decision making of chess including turn-taking and competition leading to goal-oriented positive moves (solutions) and negative problem finding experiences. This would enable multiple, repeated solving experiences to be recorded within a relatively short participation period. Tasks that produce multiple within-participant comparisons over many trials are important, particularly for experimental approaches that incorporate physiological and neuroimaging data in the study of problem solving (Bowden and Jung-Beeman, 2003b; Shen and Yuan, 2016; Hill and Kemp, unpublished b). Despite this potential, little research has focused on Connect 4. The few papers that do are from the field of Applied Computing exploring algorithms to compute the best moves to win (e.g., Allis, 1988) or to develop a learning-based computer system to play Connect 4 (Mańdziuk, 2012). Therefore, this study in addition to developing a novel methodology to elicit both positive and negative problem solving experiences further aims to explore the potential for development of computer-based Connect 4 paradigms for uses beyond Applied Computing contexts.

The first aspect necessary in developing this novel problem solving task will be to check that the experiences elicited in participants carrying out the task are those identified as relevant to the research question of interest. So in this case it will be necessary to demonstrate that a full range of solving experiences: positive and negative episodes of both insight and search are consistently reported across a range of participants and trials. As seen in the development of other problem solving paradigms (for example the CRA or magic tricks) participants are given definitions for experiences they are then asked to report having completed the task/problem (for example Jung-Beeman et al., 2004; Danek et al., 2016). A widely adopted definition given to help participants identify (positive) insight is that of Jung-Beeman et al. (2004):

A feeling of insight is a kind of ‘Aha!’ characterized by suddenness and obviousness. You may not be sure how you came up with the answer but are relatively confident that it is correct without having to mentally check it. It is as though the answer came into mind all at once-when you first thought of the word, you simply knew it was the answer. The feeling does not have to be overwhelming, but should resemble what was just described.

More recently an adapted version of this definition incorporated explicit description the alternative to insight describing analytic search as stepwise experiences, furthermore using the analogy of sudden lightbulb switching on for insight compared to gradual dimming up for search (Danek et al., 2016; Webb et al., 2016; Danek and Wiley, 2017). Yet, these studies only focus on insight as a positive experience, so a definition for this study will need to differentiate between Aha and Uh-oh experiences. However, further extending the already quite wordy definitions of insight may be problematic. Emerging evidence from qualitative work by Hill and Kemp (unpublished a) suggests that participants do not always pay attention to all aspects of the research definition of insight given. Qualitative responses were provided by participants some of which reported Uh-oh experiences that were responses to a surprising, negative external event. They appeared to ignore the given definition requiring their Uh-oh moment to be in relation to a new idea or understanding that is central to an insight moment. Furthermore, recent research has suggested that the Aha experience can be deconstructed into different dimensions and is separable from other aspects of insight solving such as solution generation (Kizilirmak et al., 2016; Danek and Wiley, 2017). For the purpose of verifying that Connect 4 elicits positive and negative experiences of insight and search solving the focus for this study is clearly on the experiential aspects of solving. Therefore the development of concise definitions should look to minimize the inclusion of material that may be distracting or less relevant and focus on the experiential components of insight and search solving.

Danek and Wiley (2017) identified three key aspects important in the experience of insight; pleasure, certainty, and suddenness. In addition they were able to demonstrate that elevated surprise ratings associated with false insight, when the participant experienced an insightful solution that was incorrect. In contrast the experience of relief was indicative of insight solutions that were correct. In Connect 4 however, each move whilst representing a solving episode, does not have a binary correct/incorrect outcome. As such surprise and relief might be less useful in delineating solving experience in this context. Likewise, a feeling of certainty may also be problematic, as there is not such a concrete outcome to judge the efficacy of a move compared to the binary question of how certain someone is that their proposed solution (for example identified word in the CRA) is correct. Therefore a focus on the remaining aspects of suddenness and pleasure (termed more broadly as emotional valence to incorporate negative experience) will be used to develop working definitions for this paradigm.

This study therefore reports the implementation of a new domain of Connect 4 in problem solving research with the aim of eliciting positive and negative, insight and search experiences reliably in participants. It will further explore the validity of this method by using established scales (feelings of insight and phenomenological ratings) used in research paradigms that focus on positive insight and search solving to measure this experience. In addition, a behavioral measure (move time) will also be compared, as this has been shown to be a distinctive aspect in previous research; with insight moves being faster than search (Kounios et al., 2008; Subramaniam et al., 2009; Danek et al., 2014b; Shen et al., 2015). As such a series of hypotheses are proposed to meet these aims. Firstly, there will be a difference in speed of moves reported for different types of solving; specifically insight moves will be faster than search. Moves labeled as positive insight and positive search will be rated as more pleasant than negative insight and search ones. Insight moves will be rated as more surprising and sudden than search. Finally, there will be no influence of solving type or valence on ratings of move certainty.

## Materials and Methods

### Participants

Eighty participants (54 female) were recruited via advertisement within the University and local community. Participants were all over 18 years old (*M*_age_ = 30.63 years, *SD*_age_ = 12.64, range _age_ 18–66 years), with a mixture of native English speakers and those with English as an additional language (*n* = 10). Some participants were repeat participants in a longitudinal study that compared solving performance across different tasks (reported elsewhere). In addition to the data reported here, additional physiological (heart rate and interoceptive heart beat counting task) and psychological measures (emotionality self-reports) were recorded (also reported elsewhere).

### Materials

A commercially developed, computer-based version of Connect 4 was used (Connect Four Fun developed by TMSOFT, tmsoft.com, copyright 2008–2016). The game has single and two player options, the former being used in this study. The ‘night’ theme was selected and used for all participants due to its relatively neutral background. In the multigame setting, the player who starts (human player or computer) is determined by the winner of the previous game, which could potentially introduce confounds, therefore a single game setting was used meaning the human player (participant) always made the first move. The level of difficulty could be selected on a game by game basis choosing from: easy, medium, hard, pro, and expert. These represented subjective labels for the difficulty of play determined by the algorithms of the game (not available to the researcher). This was not deemed to be problematic as participants were self-identifying the level to play. See below in Discussion for further evaluation of this.

### Measures

#### Feeling of Insight

Jung-Beeman et al. (2004) developed a forced choice response of either insight or non-insight. Participants made these self-report after each problem solving episode (in the original study’s case after each CRA puzzle was solved). This study adapted the self-report measure to additionally incorporate valance, creating four solving experiences as shown in Table 1. Valence was differentiated in terms of motivations for the move, positive moves focused toward winning and negative moves avoiding losing. To distinguish between insight and search, the emotional descriptors of Aha and Uh-oh were used for insight along with the key idea that these occur suddenly. In contrast, search descriptions focused on gradually working out a move. The descriptions used were consistent with previous descriptions used to explore insight (see Hill and Kemp, 2016). A further option was included in line with Bowden and Jung-Beeman (2007) who enabled participants to choose ‘other’ to ensure that participants were not forced to choose an experience that was not congruent to them. This option was labeled as neutral/or no reason.

**Table 1:** Self-reported feeling of insight: descriptions given to participants playing Connect 4.

Solving type	Description	Cue available whilst playing
Positive insight	You suddenly have an idea for your next move or how to win	Aha
Positive search	You work out your next move or how to win	I’ve worked out a good move
Negative insight	You suddenly see a problem or that you are in danger of losing	Uh-oh
Negative search	You work out a problem or that you are in danger of losing	I’ve worked out there’s a problem

#### Phenomenological Self-Report Scales

Danek et al.’s (2014a) phenomenological self-report scales were used to measure self-reported ratings of pleasantness, surprise, suddenness, and certainty of the different solution types. As detailed above this measure has been further validated in relation to an established range of insight problems by Webb et al. (2016). Impasse was not measured as participants were unlikely to experience this in the context of Connect 4 (as they would always be able to make a move and not looking for a single correct answer). Following the methodology of Danek et al. (2014a) these were presented at the end of the study after all games of Connect 4 had been played. Each VAS for phenomenological rating was presented one screen at a time in PsychoPy (Peirce, 2007, 2008) using the default VAS settings that presented the rating line in the center of the screen with labels for either end of the scale (see Table 2 for the labels for each rating scale) and prompt question above. The position marked on the line by the participant provided a score between 1 and 0. Ratings were presented in a random order in terms of both the different types of solving and rating being given. This method minimized the chance that participants were simply responding in relation to the definitions given (although does not exclude this possibility – see further in Discussion). First, as the reports were presented separately and randomized, participants’ attention was directed to the two specific aspects of each rating being requested (the solving type and phenomenological aspect being rated) reducing the likelihood of comparisons between ratings for different solving types. Second, as no numbers were used in the reports participants gave, simply a position on a line this again made it harder for participants to make reports relative to their previous ratings given.

**Table 2:** Questions asked of participants providing phenomenological ratings for the different solving types and labels for visual analog scale.

Phenomenological rating	Question	Label for extremes of VAS	
		0	1
Pleasantness	Please rate your *positive insight* experience:	Unpleasant	Pleasant
Surprise	Please rate your *negative insight* experience:	Not surprising	Surprising
Suddeness	The *negative search* idea came to me:	Slowly	Quickly
Certainty	I felt about the ideas I had through *positive search:*	Uncertain	Certain

*Italic terms changed according to type of problem participants were rating: positive insight, positive search, negative insight, or negative search.*

### Procedure

As highlighted in section Participants additional data (questionnaires and heart beat counting task) was collected before playing Connect 4, and a second heart beat counting measure was taken directly after playing and before completing the phenomenological ratings, these are reported elsewhere. The game of Connect 4 was introduced to participants both verbally and with written instructions immediately prior to playing. It was described as a game played in pairs who take turns in dropping counters in a grid with the winner being the first to get four in a row. An illustration of a Connect 4 grid with a winning game was provided and the different ways to win [horizontal, vertical, and diagonal (shown on picture) lines of four] were explained by the researcher. In addition the levels of difficulty that the game could be played at were outlined. Descriptions were then provided for the different types of solving experience in the context of playing Connect 4 (Table 1).

Participants played a practice game set to the ‘easy’ level before selecting the difficulty level they wished to play their first block of three games. Participants indicated when they had chosen their move by pressing a button on a watch (Heart Rate monitor watch) recording the time of their move decision. Participants then verbally identified their selected move (each column was labeled with a number from one to seven) and their feeling of insight when making the move. They could indicate the four solving experiences identified in Table 1 or select a neutral/no reason option. Reminders of these were provided whilst they were playing the game. The researcher recorded the experience for each move before making the move indicated, this was to avoid participants having to switch between pressing buttons on the watch and operate the Connect 4 game via the mouse or keyboard. Whilst playing the cursor was visible on the screen, therefore the researcher left the cursor in the position of the last move made (i.e., over the column of the last move) to avoid cuing the participant in any way. The participant was positioned facing the screen with the reminder sheet in front of them. They were seated next to the researcher, so no unintentional cues, such as eye movement could be detected by the participant whilst playing the game. After three games the participant had the opportunity to stay of the same level of difficulty or to change. The last three games were then played following the same protocols. The outcome of each of the six games (win, lose, or draw) was recorded by the researcher.

### Statistical Analysis

As this study includes predictions for null hypotheses, for example in relation to certainty ratings, a Bayesian approach was taken to analysis as this enables direct testing of the fit of the data to the null (H_0_) compared to alternative hypothesis (H_1_) (Jarosz and Wiley, 2014). Therefore Bayesian Repeated Measures Analysis of Variance (Bayes RM-ANOVAs) were conducted using JASP (JASP Team, 2017) to analyze main effects and interactions for solving type (independent grouping variable of insight versus search) and valence (independent grouping variable of positive versus negative) on the dependent variables of solution time and phenomenological ratings (pleasantness, surprise, certainty, and suddenness). As little previous research is available on which to produce informed priors, default priors were used with the null hypothesis assumed to have an effect size of zero while the alternative an effect size that was not zero (Rouder et al., 2009). Bayes factors are ratios that express the likelihood of alternative comparative to null hypothesis (or vice versa), they can be reported in terms of the evidence toward the alternative (BF_10_) or toward the null (BF_01_). Bayes factors of 1–3 represent weak or anecdotal evidence, between 3 and 10 as moderate, 10 and 30 as strong, and above 30 as very strong evidence toward the hypothesis indicated (i.e., BF_10_ or BF_01_) (Jeffreys, 1961; but for slightly different interpretation see Raftery, 1995). These interpretations have been adopted by researchers taking a Bayes approach within the field of experimental problem solving and insight (for overview of Bayesian approaches in the context of problem solving research see Jarosz and Wiley, 2014 and for an example of application of this analytical approach see Webb et al., 2016).

### Ethics Statement

This study was carried out in accordance with the recommendations from the University Science and Medicine Ethics Committee. All participants gave written informed consent in line with the guidelines from the British Psychological Society and in line with the Declaration of Helsinki.

## Results

**FIGURE 1:**
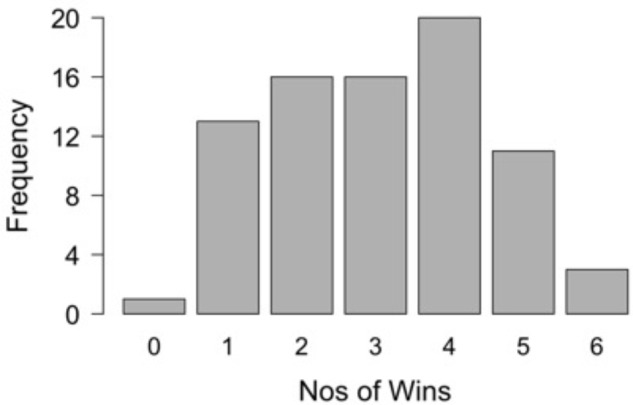
Frequency distribution of participant’s wins in Connect 4.

Participants on average won 3.1 (*SD* = 1.46) of the six Connect 4 games they played. Figure 1 shows the distribution of number of games won that approximates to being normally distributed.

### Connect 4 Frequency of Solving Types

Of all moves made, 74% were active solving experiences (search or insight rather than moves identified as neutral/no reason). 22% of these moves were insight (11% positive and 11% negative) and 78% were search (62% positive and 16%). Table 3 shows the range of solving types reported by participants whilst playing Connect 4. Just under two thirds allocated moves to all four solving types (positive insight, positive search, negative insight, and negative search) whilst over 90% experienced at least three.

**Table 3:** Breakdown of participants’ reported solving as positive insight (+i), positive search (+s), negative insight (−i), and negative search (−s).

Reported	Nos. of participants		+i	+s	−i	−s
4 Solving types	54		✓	✓	✓	✓
3 Solving types	19	6		✓	✓	✓
		1	✓		✓	✓
		5	✓	✓		✓
		7	✓	✓	✓	
2 Solving types	6	2		✓		✓
		2	✓	✓		
		2		✓	✓	
1 Solving type	1			✓		

One question of specific interest might be whether all negative insights were reported as a direct response to losing or an imminent loss of a game. Comparing negative insight reporting across all games played showed that roughly equal reporting of negative insight was seen for games that were subsequently won or drawn (41%) compared to lost (59%). Furthermore, only 14% of the total negative insight moves were for the last move in a game that was lost.

### Move Times Across Different Types of Solving

For nine participants timing data recorded on the watch was not available due to a recording fault with the equipment they were therefore excluded from analysis exploring move times. The overall mean time for a move across the remaining participants was 11.6 s (*SD* = 4.4 s). A repeated measures Bayesian ANOVA was conducted for participants who reported all four solving types (*n* = 45). Bayes factors (BF) were below three for all main effects of solving type (IV) and valence (IV) on move time (DV) and when comparing a null model incorporating the main effects to the interaction. As such this presents weak evidence of effects of solution type or valence of moves on the time taken to make them.

### Phenomenological Self-Reports

**FIGURE 2:**
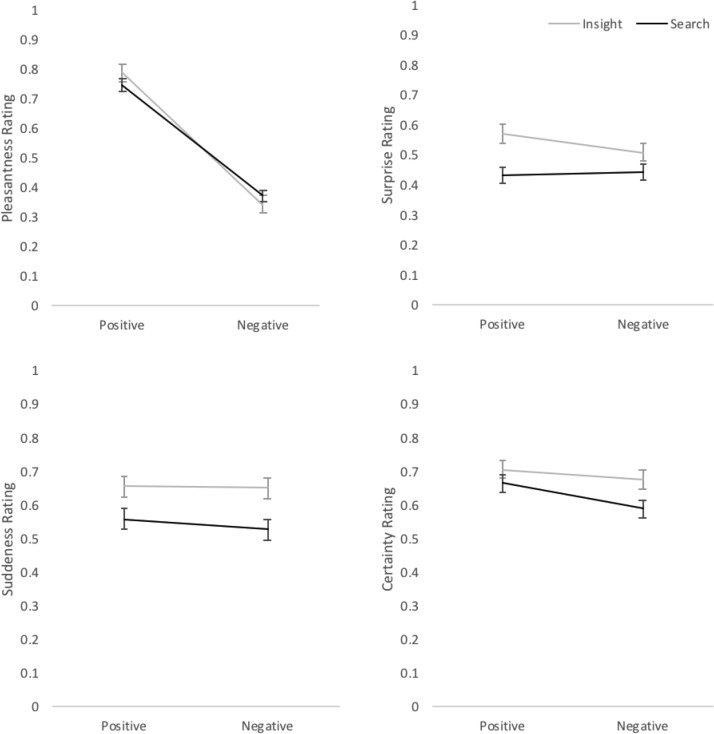
Mean phenomenological ratings for solving type (insight/search) and valence (positive/negative) for Pleasantness, Surprise, Certainty, and Suddenness. Error bars = SE.

For pleasantness ratings a repeated measures Bayesian ANOVA (IVs: Solving type and valence. DV: pleasantness) provided strong evidence of a main effect of valence (BF_10_ = 5.77e + 38) and moderate evidence of no main effect of solving type (BF_01_ = 6.88). Positive moves were rated as more pleasant than negative for both types of solving. On viewing the graph (Figure 2) presenting these findings it might appear that there was in interaction effect of solving type and valence, with insight moves rated as more positive and more negative than search. However, by adding the main effects to a null model and comparing to one with interaction effects there was seen to be weak evidence toward either model (BF = 2.35).

There was strong evidence (BF_10_ = 266.70) for a main effect of solving (IV) on surprise ratings (DV), with insight solutions being rated as more surprising than search for both positive and negative moves. There was moderate evidence of no main effect of valence (IV: BF_01_ = 3.36) or interaction effects (BF = 3.71 toward a null model including main effects compared to interaction effects) on surprise ratings.

For suddenness (DV) there was strong evidence (BF_10_ = 527.77) for a main effect of solving (IV), with insight solutions reported as more sudden than search. There was moderate evidence toward a null effect of valence (IV: BF_01_ = 5.67) and toward no interaction effects (BF = 3.57 toward the null model incorporating main effects).

For certainty ratings (DV) weak evidence was provided for all comparisons (main effects of IVs solving and valence, and interaction of the two: all BF’s < 2), meaning no conclusions could be made regarding evidence toward the null or alternative hypothesis. Graphs with ratings for the four solving types for each phenomenological scale are shown in Figure 2.

## Discussion

This study demonstrates that Connect 4 represents a naturalistic task that elicits insight and search problem solving experiences as a player make moves dropping counters into a grid, working toward the overall winning goal of getting four counters in a row. Importantly, it has demonstrated for the first time the elicitation of negative insight in a laboratory setting, meaning that validation of negative insight from an experimental perspective can be undertaken to compliment current research taking a qualitative approach (Hill and Kemp, 2016, unpublished a). The full range of solving was experienced in the majority of participants, with over 90% experiencing at least three of the four solving types. As such the utility of Connect 4 to render multiple incidences of within participant comparisons of different solving is apparent that is particularly important for experimental approaches and those that incorporate neuroimaging and physiological approaches (Bowden and Jung-Beeman, 2003b; Shen and Yuan, 2016; Hill and Kemp, unpublished b). Varying proportions of insight to search are seen for different types of elicitation task. For CRA problems around half of solved trials lead to insight reports (e.g., Jung-Beeman et al., 2004; Cranford and Moss, 2010). Magic tricks conversely gave a higher proportion of non-insight trials, ranging from 41% reported as insight by Danek et al. (2014b) to 29% by Hedne et al. (2016). It can therefore be seen that different methods elicit insight and search solutions to different degrees. Connect 4 in this study showed a lower rate of insight solving than other methods. However, whilst magic tricks and CRA paradigms provided solving experiences in under 60% of the trials, 74% of moves in Connect 4 provided reported solving experience.

Participants’ post-game phenomenological reports verified hypothesized characteristics of the experiences elicited whilst playing Connect 4 in line with previous research (Danek et al., 2014a; Webb et al., 2016), finding that positive search and insight were rated as more pleasant than negative search and insight. Furthermore showing that insight (both negative and positive) moves were experienced as more surprising and sudden. Finally, there was not sufficient evidence to support the alternative or null hypothesis exploring certainty ratings across solving and valence. As such this demonstrates that Connect 4 serves as a useful potential method to explore aspects experimentally across the full range of positive and negative insight and search solving as it performs in line with a range other insight elicitation methods that are limited to eliciting positive solving experiences.

As discussed in the Introduction, Danek et al. (2014a) identified a limitation relating to their phenomenological ratings as participants did not provide ratings for non-insight, search solutions against which to compare. Subsequent papers, however, have tended to adopt the feeling of Aha or insight measured reported on a VAS (e.g., Webb et al., 2016) again meaning comparisons between phenomenological aspects of solving experienced as insight or search was not conducted. This paper therefore offers additional support, directly testing the predictions seen in previous literature relating to aspects of pleasantness, suddenness, surprise and certainty attached to insight compared to search solving.

In terms of pleasantness, as hypothesized in this study positive insight and search solving were rated as more pleasant than negative solving. However, in previous literature it is suggested that positive emotions of happiness or pleasure were particularly associated with insight moments (Danek et al., 2014a; Shen et al., 2015). Danek et al.’s (2014a) participants, before providing the phenomenological ratings for their insight solutions also gave free reports describing their insight experiences. One of the resulting themes from this related specifically to emotional happiness, this was by far the most reported aspect relating to the insight experience. Shen et al., 2015 showed a direct comparison of happiness ratings [using different rating scales from Danek et al.’s (2014a)] for CRA insight and search solutions, showing that insight trials were rated higher for happiness than search. As such it might be predicted that positive insight would be rated as more pleasant than positive search. As little previous research has considered negative insight it is less easy to make predictions in relation to this. As shown in Figure 2, there is a pattern that suggests that positive insight might be seen as more pleasant than positive search, and negative insight be seen as more unpleasant then negative search solving. However, as highlighted by the accompanying Bayesian analysis, no definitive conclusion for or against this pattern can be reached from the current data. This is therefore something to further explore in future research.

In addition to insight being more pleasant, insight solutions are also proposed to be more sudden. Connect 4 moves labeled as insight were rated as being more sudden than search for both positive and negative solving. Danek et al. (2014a) found suddenness to be less important in insight ratings than factors of pleasantness, surprise, and certainty, but as previously mentioned did not directly compare ratings to those non-insight ratings. Shen et al. (2015) did not have a measure of suddenness but found that participants rated greater hesitation for search trials than insight, so greater hesitation would map to reduced feelings of suddenness, making this finding congruent to the current results. Corroborating behavioral findings to these perceived ratings can be seen from many early CRA studies that show faster responding for trials labeled as insight than search (e.g., Kounios et al., 2008; Subramaniam et al., 2009; Danek et al., 2014b; Shen et al., 2015 but also see critique of this by Cranford and Moss, 2010, 2011, 2012). One caution to this finding echoes that identified by Danek et al. (2014a) that suddenness formed a key part of the definition given to participants, so their ratings may simply reflect this rather than their experience of insight and search. Indeed, contrary to these self-reports there was insufficient evidence from behavioral measures of Connect 4 move speed (but see limitations below for further evaluation of this measure). Furthermore, Webb et al. (2016) highlighted that it is unclear if suddenness is an aspect of insight that generalizes across problem types. Results here would again suggest further work be necessary to be confident regarding this aspect in relation to insight compared to search in Connect 4 solving.

Previous research in the role of surprise in insight is even less clear. For example, Danek et al. (2014a) and Shen et al. (2015) found conflicting results in respect of surprise, with Shen et al. (2015) not finding that it featured in free responses participants gave in an exploratory study, whilst Danek et al. (2014a) found it was the second most important emotion after happiness. Likewise, Webb et al. (2016) demonstrated that feelings of Aha were more related to surprise than accuracy of the solution. This study again demonstrated congruent results, that insight solving was rated as more surprising than search for both positive and negative solving. Danek and Wiley (2017) suggested that surprise could further distinguish between true and false insight (where solutions were correct or incorrect), with higher surprise ratings for false insight. However, as identified in Connect 4, each move does not result in a dichotomous outcome that is either correct or incorrect, meaning such a relationship would be harder to quantify using the Connect 4 paradigm.

The absence of clear right/wrong outcomes for Connect 4 moves was again reflected in the lack of support from the data in effects for certainty ratings. Future work using the Connect 4 paradigm might consider introducing an objective measure of quality of moves that could be seen as comparable to correct/incorrect in other paradigms (e.g., Danek and Wiley, 2017). In the current study an overall marker of quality might be suggested in examining the number of games won. However, participants were able to self-select the level of difficulty they played at, meaning that the overall win rates of players were not comparable. Asking participants to play at set levels of difficulty would not make sense in terms of the aims of the study which was to elicit within participant solving experiences; if a level was too difficult or easy this would limit the solving that could take place. Figure 1 demonstrates that participants were indeed selecting a level of play of appropriate challenge, as the approximate normal distribution of winning games with no ceiling or floor effects suggests participants were not playing at a level that was too easy or difficult. Furthermore, it is the within participant efficacy of each move relating to phenomenological experience that is of interest and therefore future research should look to develop such a measure of quality of moves similar to that seen in chess research (Sigman et al., 2010). However, such a measure would require firstly all the moves made to be recorded and compared to the options on the grid at each play point, something that was not possible using the commercial version of Connect 4 employed in this study.

This highlights a current limitation of this paradigm, which is the need for a better, more fit for purpose version of Connect 4 to be developed. In addition to not being able to measure and quantify move quality the commercial version used ran a game without breaks in play. This meant that data collected whilst playing had to be done verbally requiring the presence of a researcher. Furthermore, the move time data relied on button presses on a watch which incorporated participants’ responses to the type of solving, meaning the accuracy of these is questionable. This potentially introduced confounds (although precautions were taken to minimize the experimenter effects – see Method) and for the future complete automation of the task would be desirable. For example, this study took the approach introduced by Danek et al. (2014a) of obtaining phenomenological ratings post task. More recent work has obtained these ratings for each trial of solving (see Webb et al., 2016; Danek and Wiley, 2017), which is preferable as it means the ratings are made close to the actual solving experience, minimizing memory effects and likely confounding influences of definitions on ratings obtained. In order to do so with the current Connect 4 version would require interrupting each move in the game and switching to a different software or computer to collect this data; having a bespoke Connect 4 version would enable such data collection features to be incorporated. Furthermore, heart rate data collection (reported elsewhere) that took place whilst participants played Connect 4 was compromised. There were not long enough breaks between moves to adequately ascribe heart rate effects to individual solving experiences, again adding adequate time breaks between moves is something that could be built in to a bespoke Connect 4 version.

It could be questioned if the negative insights reported in this study are true instances of negative insight or the result of negative appraisals due to losing a game. As reported in section Connect 4 Frequency of Solving Types negative insight was not only reported as a result of losing a game, with a small amount of the overall reported negative insight moves being the final move in a lost game. In fact just under half the reported negative insight moves were in winning games. This would support that participants were reporting moves reflective of their experience of problem solving rather than in response to the outcome of a game (i.e., winning or losing).

A further matter for discussion is whether the methods used in this study (and previous work in the field) simply represent circularity in relation to definitions given to participants producing corresponding phenomenological reports. However, the authors believe that several factors mitigate these concerns. Firstly, participants were not forced to choose one of the four solving types, but had the additional option of neutral/no reason. This means that if the solving descriptions given did not match participants’ experience they could indicate as such. Whilst some participants selected the no reason/neutral option for some moves, particularly early in the game (verbally for example many suggested that they always took the same first move) none exclusively selected it. This suggests the solving descriptions did map to genuine experience rather than representing a demand characteristic of a forced choice. Specifically addressing the possibility of phenomenological ratings representing demand characteristics reflecting definitions given. Firstly steps were taken to reduce this possibility (see section Phenomenological Self-Report Scales) in terms of limiting the comparisons participants could make in the ratings they provided. Furthermore, whilst definitions given did explicitly include descriptions of suddenness, they did not describe things in terms of pleasantness, surprise or certainty. Future research could further look to reduce the possibility of circularity in a number of ways. As highlighted above, a more advanced version of Connect 4 that enabled phenomenological ratings to be taken for each move made (at the time of the move rather than at the end of the study) should improve the quality of these reports. As discussed recently by Laukkonen and Tangen (2018) self-reports made as close to the solving experience as possible reduce the influence of confounds such those from memory reflecting earlier descriptions of experience given. In addition, the effect of giving definitions on subsequent phenomenological reports in problem solving paradigms could further be explored.

In summary, this study represents a proof of concept for the utility of Connect 4 as a paradigm to elicit problem solving experiences across valence (positive to negative) and solving type (insight to search). This should enable further experimental investigation of problem solving that incorporates the recently described negative insight, contrasting this to positive insight and search-based solving. Future work is required to develop better computer hosted versions of the game that would enable the incorporation of bespoke features for research designs to: minimize confounding effects such as the presence of an experimenter; enable synchronization with other equipment, for example fMRI or physiological recording and; enable within task data collection for instance as discussed above, phenomenological ratings for each move (trial).

## Data Availability

The dataset of the present study will be made available via the Open Science Framework.

## Author Contributions

All authors designed, analyzed the data, and wrote up this report. GH collected the data.

## Conflict of Interest Statement

The authors declare that the research was conducted in the absence of any commercial or financial relationships that could be construed as a potential conflict of interest.
